# Intestinal microbial metabolites in human metabolism and type 2 diabetes

**DOI:** 10.1007/s00125-020-05268-4

**Published:** 2020-09-03

**Authors:** Hilde Herrema, Jan Hendrik Niess

**Affiliations:** 1 Department of Experimental Vascular Medicine, Amsterdam University Medical Centers, Location AMC, Meibergdreef 9, 1105 AZ Amsterdam, the Netherlands; 2 Department of Biomedicine, University of Basel, Hebelstrasse 20, CH-4031 Basel, Switzerland; 3 University Center for Gastrointestinal and Liver Diseases, St Clara Hospital and University Hospital of Basel, Basel, Switzerland

**Keywords:** Intestinal barrier, Metabolites, Microbiome, Review, The metabolic syndrome, Type 2 diabetes

## Abstract

Humans with the metabolic syndrome and type 2 diabetes have an altered gut microbiome. Emerging evidence indicates that it is not only the microorganisms and their structural components, but also their metabolites that influences the host and contributes to the development of the metabolic syndrome and type 2 diabetes. Here, we discuss some of the mechanisms underlying how microbial metabolites are recognised by the host or are further processed endogenously in the context of type 2 diabetes. We discuss the possibility that gut-derived microbial metabolites fuel the development of the metabolic syndrome and type 2 diabetes.

**Electronic supplementary material:**

The online version of this article (10.1007/s00125-020-05268-4) contains a slide of the figure for download, which is available to authorised users.

**Figure:**
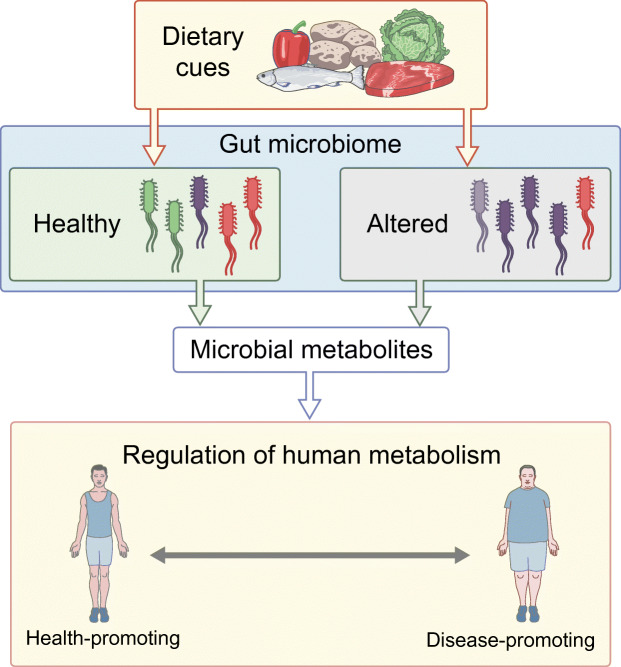
Graphical abstract

## Introduction

The gut microbiome in humans can be considered as an organ, which has functions critical for human metabolism, digestion, maintenance of gut barrier function and immunomodulation. Moreover, the gut microbiome has been linked to many diseases not classically associated with microbes, such as metabolic diseases, rheumatoid arthritis and psychiatric disorders. A skyrocketing number of publications implicate the gut microbiome in the development of metabolic diseases including obesity and type 2 diabetes. Although the majority of publications on human disease development are associative and, as yet, do not provide much mechanistic insight, the field is rapidly moving towards translational studies combining animal models with human intervention. This movement is critical to push forward our understanding of this complex community in the regulation of human metabolism.

The gut microbiome has emerged as a targetable organ with potential to alter disease development. This is important in light of the ever-increasing global prevalence of type 2 diabetes [1], which still has an unresolved clinical need for prevention and cure. This debilitating disease has high morbidity and mortality rates and is estimated to affect 700 million people by 2045 [2], with certain ethnicities being overrepresented [3–5]. Type 2 diabetes is characterised by deficient insulin secretion from the pancreatic beta cells coinciding with impaired insulin sensitivity of tissues and organs with a major role in glucose clearance (adipose tissue, liver, muscle). Many gene variants have been linked to type 2 diabetes risk [6]; these variants have been estimated to increase risk by 10–30% [7, 8]. In the vast majority of cases, however, type 2 diabetes is driven and preceded by the metabolic syndrome, a cluster of interconnected lifestyle-related clinical features that consist of elevated fasting glucose, increased BP, reduced HDL-cholesterol, increased circulating triacylglycerols and obesity. Although obesity is a critical (and noticeable) hallmark of the metabolic syndrome, other clinical features often exist unnoticed or are not present at all. The metabolic syndrome is, thus, often not recognised, making it difficult to calculate its exact global prevalence. Estimates suggest that the metabolic syndrome is three times more prevalent than type 2 diabetes [9]; this translates into 1 billion people worldwide being affected by this condition and, hence, being at risk of developing type 2 diabetes.

In addition to the metabolic syndrome being a critical risk factor for type 2 diabetes, the gut microbiome has been extensively linked with the metabolic syndrome and type 2 diabetes in humans. Although a commonly carried dogma in the field is that the gut microbiome of individuals with type 2 diabetes (or with the metabolic syndrome preceding it) is characterised by lower diversity [10–12], there are many publications that cannot confirm these data [13] (see ‘Altered microbiome composition in type 2 diabetes’ section, below).

Importantly, mouse studies [14–16] and human intervention studies [17–19] in which the gut microbiome composition is modified (e.g. by faecal microbiota transplantation [FMT]) indicate a causal role for the gut microbiome in type 2 diabetes development. Moreover, these studies imply that targeting the gut microbiome holds merit to serve as a preventive measure for type 2 diabetes development or to lower the burden on those already affected.

We would like to emphasise that the vast majority of research on the role of the gut microbiome in type 2 diabetes development is focused largely on bacteria. The gut microbiome, however, is a spectacularly complex community of microorganisms, including bacteria, fungi, protozoa and viruses (mainly bacteriophages), which, as a whole, has been implicated in the development of several human diseases. However, little is known about community-level dynamics and local interaction between members of these different kingdoms of life. This is beyond the scope of this review but anticipated by the authors to be critical for our understanding of how the gut microbiome alters human disease development and for development of microbiome-targeted interventions.

**Fig. 1:**
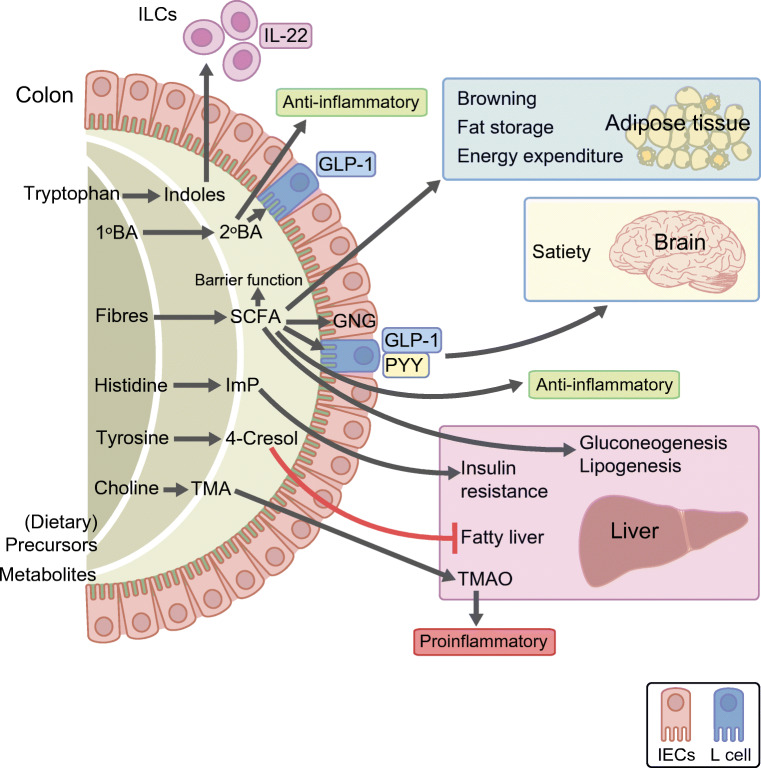
Gut-derived microbial metabolites influence host responses in the context of the metabolic syndrome and type 2 diabetes. For example, intestinal microbes metabolise diet-derived tryptophan to indoles, which in turn induce IL-22 production by ILCs [84]. In addition, primary bile acids (1ºBAs) are converted to secondary bile acids (2ºBAs) by the microbiome. Converted 2ºBAs are involved in anti-inflammatory pathways [110]. Fibres are processed into SCFAs, which facilitate browning of fat tissues, fat storage and energy expenditure in adipose tissue, satiety regulation in the brain via regulation of PYY and GLP-1 in gut neuroendocrine cells [75], and anti-inflammatory pathways in the immune system. Moreover, the SCFA butyrate is the main energy source of intestinal epithelial cells. Thereby, SCFAs help to maintain the integrity of the intestinal barrier. In addition, SCFAs promote gluconeogensis and lipogenesis in the liver. The microbiome also metabolises histidine to ImP, tyrosine to 4-cresol, and choline to TMA. ImP inhibits insulin receptor substrate signalling in the liver, and 4-cresol prevents hyperglycaemia and fatty liver in mice [98]. Meanwhile, TMA is further converted by endogenously expressed hepatic flavine-containing monooxygenase 3 into TMAO. TMAO has been implicated in inflammatory pathways relevant for the development of type 2 diabetes and for cardiovascular diseases [90]. GNG, gluconeogenesis; IEC, intestinal epithelial cells. This figure is available as a downloadable slide

With the exception of pathogenic infections or specific diseases that alter intestinal barrier function, the microbial community is generally considered to be confined to the intestine. Nevertheless, the microbiome exerts metabolic actions on most distal tissues and organs in the human body (Fig. 1). These actions are mediated by local interaction of the microbiome or microbial metabolites with immune cells, enteroendocrine cells or cells of the central nervous system. Moreover, penetrance of microbial metabolites to distal body sites has been implicated in (dys)regulation of tissue metabolic pathways. We will, in this review, focus on how microbial metabolites influence metabolic pathways relevant to type 2 diabetes development.

## Altered microbiome composition in type 2 diabetes

Several diseases are associated with a microbiome composition that is different from that in health. Although a strong definition of a healthy microbiome is lacking, the microbiome of a diseased population is generally termed ‘dysbiotic’. It must be noted, however, that the term ‘dysbiosis’ also lacks a clear definition and that ‘altered microbiome composition’ may be a more suitable term. In obesity and type 2 diabetes, microbiome composition may be altered to include overrepresentation of microorganisms (often opportunistic pathogens) that are lacking in the healthy population and reduced individual species diversity (alpha diversity). However, findings from published studies are, in part, contradictory regarding microbiome composition in individuals with type 2 diabetes, as nicely reviewed by Gurung and colleagues [20]. Since microbiome composition is regulated by many factors (in particular diet, medication use, environment and host genetic makeup), it can be challenging to determine whether the altered microbiome composition observed is a cause or consequence of a disease. As an example, metformin, an abundantly prescribed therapy for type 2 diabetes, is known to have a major impact on microbiome composition and function [21]. It is therefore of critical importance to correct for treatment regimens when studying the impact of the gut microbiome on human disease development. Moreover, because interindividual differences are large (not only in disease but also in the healthy population), it is difficult to define a health- or disease-associated microbiome (or characteristics thereof) and to reproduce findings between research groups. On a population level, large numbers (*N* > 1500) are needed to confidently identify microbiome ‘signals’ associated with variables of obesity or type 2 diabetes.

In early publications, the obesity-associated microbiome was postulated to extract more energy from dietary components when compared with healthy microbiome [15, 22]. Although some of these findings have been difficult to reproduce in other studies [23–26], several observations have been consistently and reproducibly reported. For example, proinflammatory bacteria (e.g. *Escherichia coli*) have been shown to be more abundant in type 2 diabetes [27–29]. These bacteria carry immunoreactive lipopolysaccharide (LPS) on their cell walls and are hence considered a trigger for the low-grade inflammation associated with type 2 diabetes [30–32]. Bacteria considered to have anti-inflammatory properties (e.g. *Fecalibacterium prausnitzii*), on the other hand, are generally underrepresented in the microbiome of obese humans and those with type 2 diabetes [27–29]. These anti-inflammatory properties are, in part, deduced from the capacity of these bacteria to produce the heavily studied short-chain fatty acid (SCFA) metabolites. SCFAs (primarily acetate, propionate and butyrate, which are present in the colonic lumen in a molar ratio of 3:1:1) are produced, on a large scale, from the microbial fermentation of fibres, a process estimated to provide 5–10% of our daily energy. Locally, intestinal epithelial cells have been estimated to derive 70% of their energy from butyrate, which is mainly produced by bacteria with proposed beneficial effects for host metabolism (e.g. *Ruminococcus* and *Faecalibacterium*) in mice [33]. It is important to mention, however, that the vast majority of potentially pathogenic bacteria also have the machinery to produce SCFAs [34]. Whether or not this translates into actual use of this capacity (i.e. leading to SCFA production) depends on substrate availability and local circumstances. Moreover, certain strains might have increased response to substrates when compared with close relatives, as shown for *Bifidobacterium pseudocatenulatum* [21]. Interestingly, although SCFA producers have been reported to be relatively depleted in obesity and type 2 diabetes, SCFA levels have been reported to be increased in a diet-dependent fashion [26, 35–37]. Deficient SCFA handling in type 2 diabetes has been postulated but this hypothesis remains to be confirmed. This path is likely paved with challenges as SCFA turnover is difficult to assess due to the above-mentioned utilisation of SCFA by intestinal cells as well as bacteria. In the vast majority of studies, a snapshot of the SCFA levels at a defined moment in time is provided. Such data are unsuitable readouts for SCFA metabolism.

## The intestine as a critical firewall for microbial metabolite production, passage and signalling

The microbiome of the gastrointestinal tract develops when a baby’s immune system is exposed to microorganisms, fungi and viruses after birth [38]. This community stabilises around 1 year after birth, after weaning and when a regular diet has been introduced [39]. From the beginning of life, an intimate relationship between the host and the microbiome develops [40]. The microbiome and host have a two-way relationship in which the host provides an ecological niche for the microorganisms, and the microbiome supports the host in digestion and uptake of vitamins [41, 42], bile acids and amino acids [43], development of the immune system [44, 45] and in protection from invading pathogens [46]. Functional metagenomics, an experimental approach to study the function of encoded proteins, may be used to identify bacterial effector genes and gene products from the gut microbiome that mimic endogenously produced metabolites by the host. For example, microbial commendamide (an *N*-acyl-3-hydroxyglycine) resembles endogenously produced *N*-acyl-amide signalling molecules. Thereby, *N*-acyl-3-hydroxyglycines signal through the G protein-coupled receptor (GPCR) G2A/132 and suppress proinflammatory immune responses [47]. To avoid unwanted inflammatory responses to the symbiotic microorganism, the host has developed strategies to reduce contact between the immune system and microorganisms [48]. These firewalls include the mucous separating the microorganism from the epithelial layer sealed by tight junctions [49], production of antimicrobial peptides by Paneth cells [50], macrophages located beneath the epithelium to ingest and destroy breaching (pathogenic) bacteria [51], and production of IgA [52]. IgA facilitates immune exclusion, a host defence system that prevents interaction of antigens with epithelial cells, by attenuation of bacterial motility, growth and adhesion to epithelial cells [53]. It also facilitates immune inclusion by promoting the growth of beneficial bacteria while inhibiting potential harmful competitors of these beneficial bacteria in the same niche [52, 54]. In rare circumstances, after breakdown of these firewalls, symbiotic microorganisms disseminate through blood vessels or the portal vein and are killed by macrophages in the spleen and Kupffer cells in the liver. Individuals with liver cirrhosis as a possible consequence of non-alcoholic fatty liver disease (NAFLD) exhibit impaired clearance of disseminated microbes in the liver and, thereby, have a high risk of developing systemic infections [55]. In humans with the metabolic syndrome, altered microbiome composition together with a defective intestinal barrier has been suggested to facilitate translocation of microbes, thereby contributing to low-grade inflammation. Although bacterial DNA has indeed been reported in circulation and tissues of people with type 2 diabetes, the extent to which living bacteria translocate from the gut to other body sites in humans is the topic of extensive debate [56, 57]. Since technical challenges currently do not allow us to fully clarify this question, the concept of bacterial translocation remains controversial.

Bacteria-derived metabolites, often with hormone-like activity, are more likely to disseminate into the circulation [58]. LPS, a cell-wall component of Gram-negative bacteria, and flagellin, a structural component of the bacterial locomotor appendage flagellum, are particularly recognised for their immunomodulatory properties. LPS levels are increased in humans with type 2 diabetes and in mouse models of the metabolic syndrome, a phenomenon known as metabolic endotoxaemia [31, 32]. In addition, administration of LPS was found to reduce glucose tolerance and glucose-stimulated insulin secretion in mice, in which activation of inflammatory pathways is considered a central mechanism in the development of the disease phenotype. LPS and flagellin are recognised by Toll-like receptors 4 and 5, respectively. These pattern-recognition receptors are mainly expressed on epithelial cells and cells of the innate immune system and play critical roles in activation of the immune response.

## Gut microbe-derived metabolites

In addition to microbes or their structural components, intestinal microbial metabolites also enter the host; approximately 10% of all metabolites in the blood of mice stems from the microbiome [59]. High-throughput metabolomics strategies comprising mass spectrometry coupled with gas- or liquid-phase chromatography and nuclear magnetic resonance spectroscopy provide a snapshot of the metabolome in people with type 2 diabetes. These strategies have revealed novel associations between microbial or endogenous metabolites (most notably sugars, lipids and amino acids) and variables of type 2 diabetes or the metabolic syndrome that precedes it [60]. Insulin resistance and risk of developing type 2 diabetes have been strongly associated with branched-chain amino acids (BCAAs) and aromatic amino acids, respectively. In addition, levels of gluconeogenic precursors and lipids, such as sphingomyelins, triacylglycerols and palmitate, are generally increased in people at risk of developing type 2 diabetes. Mechanistically, amino acids directly impair insulin signalling in skeletal muscle and reduce glucose uptake. BCAAs have been implicated in short- and long-term regulation of insulin secretion by pancreatic beta cells and are reported to prohibit beta cell function by inducing hypersecretion, an important hallmark of type 2 diabetes [61, 62].

Although metabolites are small intermediate- or end-products of chemical processes that keep cells alive, it is important to note that the fairly high rate of bacterial cell death in the intestine also contributes to the metabolite load [63, 64]. Gut microbial metabolites mostly stem from dietary components fermented by bacteria, such as SCFAs (mainly butyrate, acetate, propionate), unsaturated and saturated medium- and long-chain fatty acids (LCFAs), and tryptophan metabolites. Microorganisms also modify endogenous metabolites, such as bile acids, intermediates of the citric acid cycle and cholesterol metabolites, and bacteria can de novo synthesise metabolites, such as adenosine triphosphate. The penetration of host tissues by these gut microbial metabolites is classically demonstrated by comparing metabolite profiles of germ-free rodents (which lack microbiome) with metabolite profiles of conventional rodents [65–67]. When using this approach, it is assumed that metabolites that are absent in germ-free rodents are derived from the microbiome; however, this approach does not distinguish between metabolites that can be produced by both the microbiome and the host. More importantly, differences between the concentrations of these ‘overlapping’ metabolites in germ-free and control rodents might be owing to the fact that the absence of microbiome is likely to alter endogenous metabolite production in germ-free animals. Uchimura and co-workers used an elegant approach to show metabolite penetration into tissues of germ-free mice [68]. To this end, mice were treated with *E. coli* labelled with ^13^C (which makes up only 1.1% of all naturally occurring carbon), which generate ^13^C-labelled metabolites. In contrast, all metabolites produced by the mouse are ^12^C, which accounts for approximately 99% of natural carbon. ^13^C-labelled *E. coli*-derived metabolites could hence be discriminated from ^12^C mouse metabolites. Bacterial amino acids penetrated almost all organs assessed, whereas bacterial lipids mainly appeared in the intestinal regions. The proximal small intestine was the main site for microbial metabolite uptake.

Of importance, there is limited information on small-intestinal microbial metabolite metabolism in humans. Only one study has been reported in participants with the metabolic syndrome; these individuals were receiving an FMT and duodenal biopsies were collected [17]. This may be because the upper part of the small intestine, the duodenum, can only be accessed by oesophagogastroduodenoscopy, which allows the inspection of the first portion of the small intestine (duodenum). The distal part of the small intestine, the terminal ileum, can only be accessed by ileocolonoscopy. Ileocolonoscopy requires colon cleansing, which tremendously affects the composition of the microbiome and probably the metabolome produced by the gut microbiome. The 3 to 5 m between the duodenum and the terminal ileum (the jejunum) are not accessible by oesophagogastroduodenoscopy or ileocolonoscopy and are, hence, understudied.

Intestinal barrier function (e.g. mucus and IgA production) is mainly matured by the gut microbiome [69]; hence, germ-free mice have deficient gut barrier function. To correct for this in one study, germ-free mice were transiently colonised to mature the gut barrier [70]. To address the role of immunoglobulins in gut barrier function, microbial metabolite penetration was compared in transiently colonised germ-free wild-type mice and *Igh-J*-knockout mice, which lacked all antibody isotypes (IgA, IgG, IgM, IgD). Microbial metabolite levels were higher in the distal intestinal contents of conditioned wild-type mice when compared with conditioned *Igh-J*-knockout mice. In line with the hypothesis that IgA reduces the dwell time of bacteria and their metabolites in the small intestine, there was greater uptake of metabolites in the small intestine of *Igh-J*-knockout mice and unconditioned germ-free mice compared with conditioned germ-free mice. Of importance, increased exposure of host organs to microbial metabolites enhanced activation of the immune system. Increased low-grade inflammation, potentially via the gut microbiome, is an event strongly related to diabetes development [30–32]. Thereby, the induction of IgA facilitates the transit of intestinal bacteria from the small intestine into the colon. Bacterial metabolites penetrate into the host mainly in the small and not large intestine. Thereby, the transit of bacteria into the colons prevents excessive accumulation of bacterial metabolites in the host.

## Gut microbe-derived metabolites alter endogenous metabolism

### Metabolite-sensing receptors

After their penetration into the host, microbial metabolites are further processed by endogenous enzymes. Although GPCRs have been described to directly recognise bacterial metabolites in host tissues [71, 72], the majority of ‘metabolite-sensing receptors’ are yet to be identified (Table 1). Examples of metabolite-sensing receptors include the SCFA receptors GPR41 (free fatty acid receptor [FFAR]3), GPR43 (FFAR2) and GPR109A (niacin receptor 1 [NACR1]) that mediate a wide range of effects on host metabolism. These receptors, both separately and in concert, have been implicated in the regulation of satiety [73], browning of white adipose tissue and fat accumulation [74], glucagon-like peptide 1 (GLP-1) secretion [75], intestinal gluconeogenesis and peptide YY (PYY) secretion [76]. SCFAs also inhibit the activity of histone deacetylase, leading to histone hyperacetylation, by which the expression of approximately 2% of all mammalian genes are repressed [77]. Multiple receptors bind SCFAs and a given SCFA may bind to several receptors. In addition to their signalling properties, SCFAs have been shown to serve as gluconeogenic and cholesterogenic precursors in mice [78] and lean humans [79] thereby also serving a quantitative role as catabolic or anabolic substrates. Basic and clinical studies are required to dissect this system further before therapies targeting the SCFA receptors can be developed.

**Table 1:** Gut microbiota-derived metabolites associated with the metabolic syndrome/type 2 diabetes

Metabolite	Receptor	Cells expressing receptor	Effect relevant to the metabolic syndrome/type 2 diabetes	Reference
SCFAs				
Butyrate, acetate, propionate, valerate	GPR41 (FFAR3)	Pancreatic beta cells, adipocytes, enteroendocrine cells, mononuclear cells, macrophages	Inhibition of insulin secretion	Tang et al [132]
Butyrate, acetate, propionate, valerate	GPR43 (FFAR2)	Pancreatic beta cells, adipocytes enteroendocrine cells, neutrophils, eosinophils	Inhibition of insulin secretion	Tang et al [132]
Butyrate and nicotinic acid (niacin)	GPR109A (NACR1)	Adipocytes, hepatocytes, intestinal epithelial cells, neutrophils, dendritic cells, macrophages	Microencapsulated delayed niacin-release system improves insulin sensitivity in humans; GPR109A activation reduces NEFA release from adipocytes	Fangmann et al [133]; Tunaru et al [134]
MCFAs and LCFA				
MCFAs and LCFA	GPR40 (FFAR1)	Pancreatic beta cells, enteroendocrine cells	Insulin release	Itoh et al [135]
MCFAs	GPR120 (FFAR4)	Adipocytes, neutrophils, macrophages	Release of GLP-1; development of obesity and glucose intolerance in adipose tissue of HFD-fed *Gpr120*^−/−^ (also known as *Ffar4*^−/−^) animals	Hirsawa et al [136]; Ichimura et al [137]
Commendamide [*N*-(3-hydroxy-1-oxohexadecyl)-glycine]	GPR132 (G2A)	Monocytes, macrophages, granulocytes, T cells	Suppression of proinflammatory immune responses	Cohen at al [47]
Lysophosphatidic and palmoic acid	GPR35	Macrophages, monocytes, dendritic cells, neutrophils, mast cells, intestinal epithelial cells, neurons, adipocytes	Stimulation of lipid metabolism, and thermogenic and anti-inflammatory gene expression	Agudelo et al [138]
Amino acids				
Tryptophan-derived indoles	AhR	Monocytes, macrophages, ILCs, T cells, dendritic cells	Prevention of obesity and liver steatosis in mice treated with an AhR antagonist	Rojas et al [139]
Amine oxides				
TMA (further converted into TMAO by the liver)	No receptor identified yet	–	Macrophage cholesterol accumulation and associations with cardiovascular diseases; decrease in reverse cholesterol transportation	Koeth et al [90]; Wang et al [93]
Azoles				
ImP	No receptor identified yet	–	Inhibition of hepatic insulin signalling	Koh et al [98]
Cresols/methylphenols				
4-Cresol	No receptor identified yet	–	Prevention of hyperglycaemia and fatty liver disease and stimulation of insulin secretion	Brial et al [99]
Bile acids				
Lithocholic acid, taurolithocholic acid	TGR5 (GPR131)	Adipocytes, muscle, monocytes, macrophages	Increased energy expenditure, thereby preventing obesity and insulin resistance	Watanabe et al [110]
Chenodeoxycholic acid	FXR-α (NR1H4)	Small intestine, liver	Repression of bile acid synthesis; regulation of cholesterol and triacylglycerol synthesis	Makishima et al [140]; Cariou et al [141]

HFD, high-fat diet; MCFA, medium-chain fatty acid; NACR1, niacin receptor 1; NR1H4, nuclear receptor subfamily 1 group H member 4

Another metabolite-sensing receptor, the transcription factor aryl hydrocarbon receptor (AhR), senses xenobiotic chemicals, such as aromatic aryl hydrocarbons [80]. Recent work suggests that the AhR also recognises derivatives of the essential aromatic amino acid tryptophan [81]. Two endogenous pathways (the kynurenine and serotonin pathways) and one microbe-dependent pathway (the indole pathway) are involved in the metabolism of diet-derived tryptophan. In the kynurenine pathway, tryptophan is metabolised to kynurenine and kynurenic acid, while in the serotonin pathway, tryptophan is metabolised to the neurotransmitter serotonin. In the indole pathway, gut-resident microbes use tryptophan as a nitrogen source and, thereby, also produce indoles, which bind to the AhR [82]. The AhR facilitates several functions in the gastrointestinal tract, such as regulation of peristalsis and motility [83] and induction of IL-22 production by T cells and innate lymphoid cells (ILCs) [84]. Interestingly, in mice, IL-22 production by ILCs can be inhibited by vasoactive intestinal peptide (VIP)-expressing enteric neurons, thereby linking the immune and nervous systems in the gut [85]. As food consumption elicits VIP production by neurons, these VIP^+^ neurons inhibit IL-22 production by ILCs during food intake, which is associated with reduced production of antimicrobial peptides and with overgrowth of potentially pathogenic bacteria, such as segmented filamentous bacteria in mice. In contrast, feeding induces expression of lipid-binding proteins and transporters. Thus, the recognition of tryptophan-derived metabolites by AhRs regulates gut motility, pathobiont overgrowth and lipid uptake by inhibition of IL-22 production through VIP-expressing enteric neurons during nutrient uptake [85].

Many bacteria are also capable of producing mammalian neurotransmitters, such as dopamine, noradrenaline (norepinephrine), serotonin or γ-aminobutyric acid (GABA) [86]. Although it remains to be shown whether microbial production of some of these neurotransmitters indeed alter endogenous metabolism, some have been shown to activate the gut–brain axis and have been implicated in the modulation of endogenous metabolism. GABA, for example, has long been known to be produced by the gut microbiome, including members of the *Bifidobacterium* and *Lactobacillus* genera [87]. It has been implicated in glucose homeostasis and has been shown to improve beta cell function [88, 89]. Of interest, GABA was the most altered metabolite in obese humans who received an allogenic faecal transplant from lean donors [17]. This finding was associated with improved insulin sensitivity. Altogether, nutrition, in concert with the gut microbiome and the host, influences intestinal motility and the enteroendocrine system.

### Endogenous enzymes

Alongside the recognition of microbial metabolites by metabolite-sensing receptors, endogenously expressed enzymes further process microbial metabolites to influence host responses to microbial metabolites. Trimethylamineoxide (TMAO) is the most-studied diet-derived microbial metabolite and has been shown to have a strong association with cardiovascular disease development [90]. Dietary choline and carnitine can be converted into trimethylamine (TMA) by TMA-lyase-expressing bacteria in the gut. TMA is converted into TMAO in the liver by flavine-containing monooxygenase 3 (FMO3). TMAO has been linked to cardiovascular disease development in humans and mice via mechanisms that include blood platelet hyperresponsiveness [91, 92], decreased reverse cholesterol transport [90] and macrophage cholesterol accumulation [93]. Although TMAO has been implicated in the regulation of inflammatory pathways [94, 95] and endoplasmic reticulum stress [96] (both of relevance for type 2 diabetes development), evidence for a role of TMAO in type 2 diabetes is scarce [97]. Nonetheless, considering the fact that most people with type 2 diabetes will develop cardiovascular disease, we assume that TMAO might also play a role in type 2 diabetes.

The histidine derivative imidazole propionate (ImP), which is produced by microbes, is of specific interest in relation to type 2 diabetes as it was found to be positively correlated with insulin resistance in humans [98]. Although a receptor has not yet been identified, ImP was shown to directly inhibit hepatic insulin receptor substrate signalling in mice, with p38 mitogen-activated protein kinase (MAPK)-mediated hyperactivation of the mechanistic target of rapamycin complex 1 (mTORC1) being identified as a central regulatory mechanism [98]. In a recent study in humans on the extreme end of cardiometabolic disease (obese and hyperglycaemic), the microbial metabolite 4-cresol negatively correlated with type 2 diabetes [99]. Although 4-cresol can be derived directly from food, it is also a product of tyrosine and phenylalanine fermentation in the colon. Subcutaneous administration of 4-cresol prevented development of hyperglycaemia and fatty liver in mice fed a high-fat diet [99]. In line with observations that 4-cresol increases insulin secretion, this metabolite downregulated the expression of dual-specificity tyrosine-regulated kinase-1a (DYRK1A) [99], which has been shown to facilitate pancreatic beta cell proliferation [100]. In addition, *Lactobacillus plantarum*, *Enterococcus faecalis* and *Bacteroides thetaiotaomicron* produce LCFAs, including conjugated linoleic acids (CLAs) [101]. LCFAs serve as precursors for lipid mediators such as arachidonic acid, which has been implicated in metabolic improvement in humans [102]. CLA was shown to alter fatty acid metabolism and reduce body fat content in humans with obesity [103].

### Endogenous metabolites

The host also secretes endogenously generated metabolites into the intestine, where the microbiome further modifies these products. Bile acids are an important class of endogenous molecules required for the digestion of dietary fats and oils, absorption of fat-soluble vitamins, and elimination of cholesterol and bilirubin that undergo microbial conversion in the large intestine. Moreover, bile acids have long been implicated in the regulation of human metabolism and development of metabolic diseases including type 2 diabetes [104]. Bile acids are produced from cholesterol in the liver and secreted as primary bile acids in the small intestine where they aid in absorption of dietary lipids and vitamins. Although the vast majority of bile acids are reabsorbed in the distal small intestine, a small amount ends up in the colon where, depending on microbiome composition, they are converted into secondary or tertiary bile acids [105, 106]. These hydrophobic molecules easily diffuse into the circulation to bind to a broad range of receptors. The signalling properties of bile acids of relevance for type 2 diabetes are, in large, mediated via the G protein-linked receptor TGR5 and the farnesoid X receptor (FXR), both of which have high affinity for hydrophobic bile acids. In mice, activation of TGR5 by bile acids has been shown to induce GLP-1 secretion [107], to be immunosuppressive [108] and to increase energy expenditure [109, 110]. Regulation of metabolism by FXR is marked by contradicting findings since bile acids can serve as either FXR agonists or antagonists. In addition, differential regulation of FXR signalling in tissues with high expression of this nuclear receptor (most notably intestine and liver) is likely to account for the discrepancies in the field. Both activation [111, 112] and inhibition [113–115] of FXR has been shown to have beneficial effects on obesity and insulin resistance. Of importance, most of the insights into the role of FXR in the regulation of metabolism have been derived from studies in mice, which have a vastly different bile acid composition to humans [104].

Taken together, food-, host- and microbe-derived metabolites influence multiple processes required for intestinal immunity, intestinal motility and maintenance of the enteroendocrine system, all of which may be affected in individuals with the metabolic syndrome.

## Possible interventions

### Diet

Since dietary components are used by gut microbes to produce metabolites, dietary interventions are an interesting non-pharmacological approach to altering (potentially harmful) microbial metabolites, with the aim of benefitting individuals with the metabolic syndrome or type 2 diabetes. Dietary interventions are, however, often challenged by limited adherence. In addition, the so-called substitution effect, where alterations in macronutrient composition are usually inevitable in order to establish isoenergetic diets [116], can be a critical confounding factor. This ‘swapping’ of macronutrients can have vast consequences for the microbiome or can directly alter human metabolism to blur effects mediated by the microbiome. It is too early to tell whether dietary strategies aimed at reducing precursors (e.g. choline, histidine) of potentially harmful metabolites (e.g. TMAO, ImP) will indeed lead to reduced development of type 2 diabetes in the long term. Targeting bacterial enzymes, such as bile salt hydrolase (responsible for conversion of primary to secondary bile acids) or TMA lyase (responsible for conversion of choline to TMA), might hold great potential.

Importantly, dietary interventions are generally characterised by a highly personal response (i.e. success of diet) [10, 117, 118]. Interestingly, it has been shown that gut microbiome data from individuals with the metabolic syndrome with differential postprandial glucose responses can be used to generate a machine learning algorithm that can predict the individual response to and success of a particular dietary regimen in a separate cohort, based on their baseline microbiome composition [119]. Although metabolites were not addressed in this study, a similar approach can be used to develop dietary strategies best suited to alter metabolite production based on baseline microbiome genetic potential. In a study by Zhao and co-workers, participants with type 2 diabetes were administered large amounts of dietary fibres [120]. Using a metagenomics approach, gut microbiome responses was measured and linked to glucometabolic response. Participants that benefited from a high-fibre diet (e.g. who had improved HbA_1c_ and reduced body weight) and had increased gut SCFA concentrations were characterised as having increased abundance of specific SCFA-producing bacteria and coincided with increased faecal SCFA levels after the intervention. These study findings strongly suggest that dietary intervention studies aimed at altering microbial metabolite production can be successful but only when using a personal approach based on baseline microbial potential. In addition, they might explain, in part, the many discrepancies observed in the success of dietary interventions.

### SCFAs

Direct supplementation with SCFAs has thus far not yielded overwhelming (perhaps anticipated based on mouse data) evidence for improvements in glucometabolic variables in humans with the metabolic syndrome or type 2 diabetes. One study in obese individuals showed that colonic infusion of propionate stimulated release of PYY and GLP-1 and reduced energy intake, potentially via regulation of satiety [121]. In addition, insulin sensitivity in participants receiving propionate stabilised, whereas deterioration was observed in those in the control group. Acetate infusion into the distal colon was shown to increase fatty acid oxidation and PYY release while also increasing postprandial glucose and insulin levels [122]. However, supplementation with sodium butyrate for 1 month did not alter hepatic or peripheral insulin sensitivity in men with the metabolic syndrome [123]. Of interest, butyrate did improve insulin sensitivity in lean participants. This effect was attributed to putative differences in intestinal SCFA handling in obese vs lean humans, although this hypothesis remains to be tested in well-designed (stable isotope) studies in participants with type 2 diabetes. As pointed out earlier, care should be taken when interpreting studies involving SCFAs in humans since SCFA ‘faith’ and turnover is difficult to measure. An important basis for much needed clinical trials addressing SCFA fluxes in the metabolic syndrome and type 2 diabetes was highlighted by Boets et al [124]. Systemic availability and conversion of SCFA was quantified in lean participants receiving colon-delivery capsules containing ^13^C-labelled SCFA. In the circulation, 36%, 9% and 2% of administered acetate, propionate and butyrate, respectively, was detected. In addition, a major proportion of acetate (24%) was converted into butyrate or incorporated into cholesterol (1%) and fatty acids (15%). An estimated 6% of propionate was incorporated into glucose. Faecal SCFA concentrations varied greatly between individuals, as observed in several other studies in humans [125, 126], potentially due to differences in microbial genetic capacity, which was not addressed in this study.

### Bile acids

Given the many effects of bile acids on the characteristics of the metabolic syndrome and type 2 diabetes, altering human metabolism via bile acids or bile acid receptor-mediated signalling is an appealing, yet complicated, approach. As indicated, there are vast differences between mouse and human bile acid composition. Hence, data from mouse studies are difficult to extrapolate to humans. Strategies for altering bile acid composition/signalling via the gut microbiome in the context of the metabolic syndrome or type 2 diabetes are, to the best of our knowledge, scarce. Ursodeoxycholic acid (also known as ursodiol [UDCA]) was shown to improve metabolism in mice fed a high-fat diet [127]. Because UDCA is approved by the US Food and Drug Administration as a safe treatment option for primary biliary cholangitis, it might be a safe treatment option for the metabolic syndrome. A clinical trial using oral UDCA in humans is currently ongoing (ClinicalTrials.gov registration no. NCT02033876).

Furthermore, in an interim analysis of an ongoing multicentre, randomised, double-blinded, placebo-controlled trial, the FXR agonist obeticholic acid (25 mg) improved fibrosis and non-alcoholic steatohepatitis (NASH) disease activity in 23% of participants with F1–F3 fibrosis assessed by histological scoring. Fifty-one per cent of the participants with NASH receiving obeticholic acid developed pruritus, which may hinder the usage of obeticholic acid in the majority of people with NASH [128].

### FMT

FMT involves the transfer of the faecal microbiota from a healthy, screened donor to a recipient. FMT is currently only approved as a treatment strategy for recurrent *Clostridium difficile* infections and not for the treatment of the metabolic syndrome or type 2 diabetes. Despite several challenges with current FMT protocols, such as standardisation and preparation of the transplant and the route of administration, FMT can be a useful research tool to study the effect of gut microbiome and microbial metabolites on human metabolism [129]. FMT leads to major (albeit temporary in a significant number of recipients) changes in microbiome composition. Although metabolite data are not generally available in FMT studies in recipients with type 2 diabetes, it is plausible that metabolite production is also altered. One study showed that improvements in insulin sensitivity coincided with changes in beneficial microbes and microbial metabolites in insulin-resistant recipients [17]. FMT can, therefore, serve an important role in identification (mining) of novel strains and metabolites to be further studied in relevant model systems and clinical trials. This field is in the early stages and microbial metabolites, with the exception of SCFA, have not yet entered clinical trials.

FMT has been used as a useful research tool to identify novel bacterial strains. For example, in one study the butyrogenic strain *Anaerobutyricum soehngenii* was identified to positively correlate with improved insulin sensitivity after FMT [18]. In a recently published phase I/II trial in humans with the metabolic syndrome, supplementation with *A. soehngenii* was shown to be safe and to improve insulin sensitivity after 4 weeks of daily treatment [130]. Although no effect on faecal SCFA levels was observed in the given setting, trials like these will open up more treatment opportunities using microbial metabolites. It is possible that in the future it will be sufficient to supplement humans with the metabolic syndrome or type 2 diabetes with defined microbes or metabolites instead of transplanting the entire microbiome. In a randomised, placebo-controlled proof-of-concept exploratory study, *Akkermansia muciniphilia* supplementation for 3 months improved insulin sensitivity, reduced plasma cholesterol and decreased body weight [131].

## Future perspectives

Increasing evidence suggests that not only microbes and their structural components but also their metabolites influence host responses and may contribute to the development of the metabolic syndrome and type 2 diabetes. A better understanding of the relationship between the microbiome (and its metabolites) with the host will increase our knowledge on the development of the metabolic syndrome and type 2 diabetes. This is also likely to give insight into how the development of the metabolic syndrome and type 2 diabetes can be prevented and will pave the way to novel approaches for the treatment of type 2 diabetes. Before this fascinating research enters daily practice, we anticipate the following frontiers that need to be further studied.

1. Increasing numbers of studies show an association of an altered gut microbiome and its metabolome with the metabolic syndrome/type 2 diabetes. Currently the field is strongly moving towards causal/mechanistic studies, in which individual microbes or metabolites will be identified that may contribute to the metabolic syndrome/type 2 diabetes. Ideally, these studies will integrate host genetics to characterise the relationship between the microbiome and the host in the contexts of the metabolic syndrome/type 2 diabetes.
2. The characterisation of metabolite-sensing receptors and their microbial ligands will provide new options for pharmacological interventions for the metabolic syndrome and type 2 diabetes. Before metabolite-sensing receptors such as GPCRs can be targeted by small molecules, the characterisation of ligand specificity of each unique receptor is required, as most ligands/metabolites are promiscuous and bind to different receptors. Collaborations between academic investigators and the pharmaceutical industry may solve these issues.
3. Because dietary interventions are characterised by personal responses, microbiome data together with glucose response data will predict, via machine learning algorithms, individual response to interventions in personalised medicine approaches in the near future. This will require interdisciplinary teams including microbiologists/molecular biologists, bioinformaticians, endocrinologists, gastroenterologists and nutritional counselling. It is likely that only large and dedicated institutions can provide such an environment, which may hinder the introduction of personalised nutrition in daily practice.

With this knowledge, in the future it will be possible to move the fascinating microbiome research into the clinic, beyond only studying associations between the microbiome and the metabolic syndrome/type 2 diabetes. The altered microbiome in the metabolic syndrome/type 2 diabetes might be corrected in future to treat individuals with the metabolic syndrome/type 2 diabetes.

## Electronic supplementary material

Figure slide (PPTX 283 kb)

## Abbreviations

AhR: Aryl hydrocarbon receptor
BCAA: Branched-chain amino acid
CLA: Conjugated linoleic acid
FFAR: Free fatty acid receptor
FMT: Faecal microbiota transplantation
FXR: Farnesoid X receptor
GABA: γ-Aminobutyric acid
GLP-1: Glucagon-like peptide 1
GPCR: G protein-coupled receptor
ILC: Innate lymphoid cell
ImP: Imidazole propionate
LCFA: Long-chain fatty acid
LPS: Lipopolysaccharide
NASH: Non-alcoholic steatohepatitis
PYY: Peptide YY
SCFA: Short-chain fatty acid
TMA: Trimethylamine
TMAO: Trimethylamineoxide
UDCA: Ursodeoxycholic acid
VIP: Vasoactive intestinal peptide
