# Supplementary material for: Intestinal microbial metabolites in human metabolism and type 2 diabetes
Source: Diabetologia. 2020 Sep 3;63(12):2533–47. doi: 10.1007/s00125-020-05268-4 (PMC7641949; doi:10.1007/s00125-020-05268-4)
Supplement: Supplementary file 1 — (PPTX 283 kb) [file 125_2020_5268_MOESM1_ESM.pptx]

## Slide 1
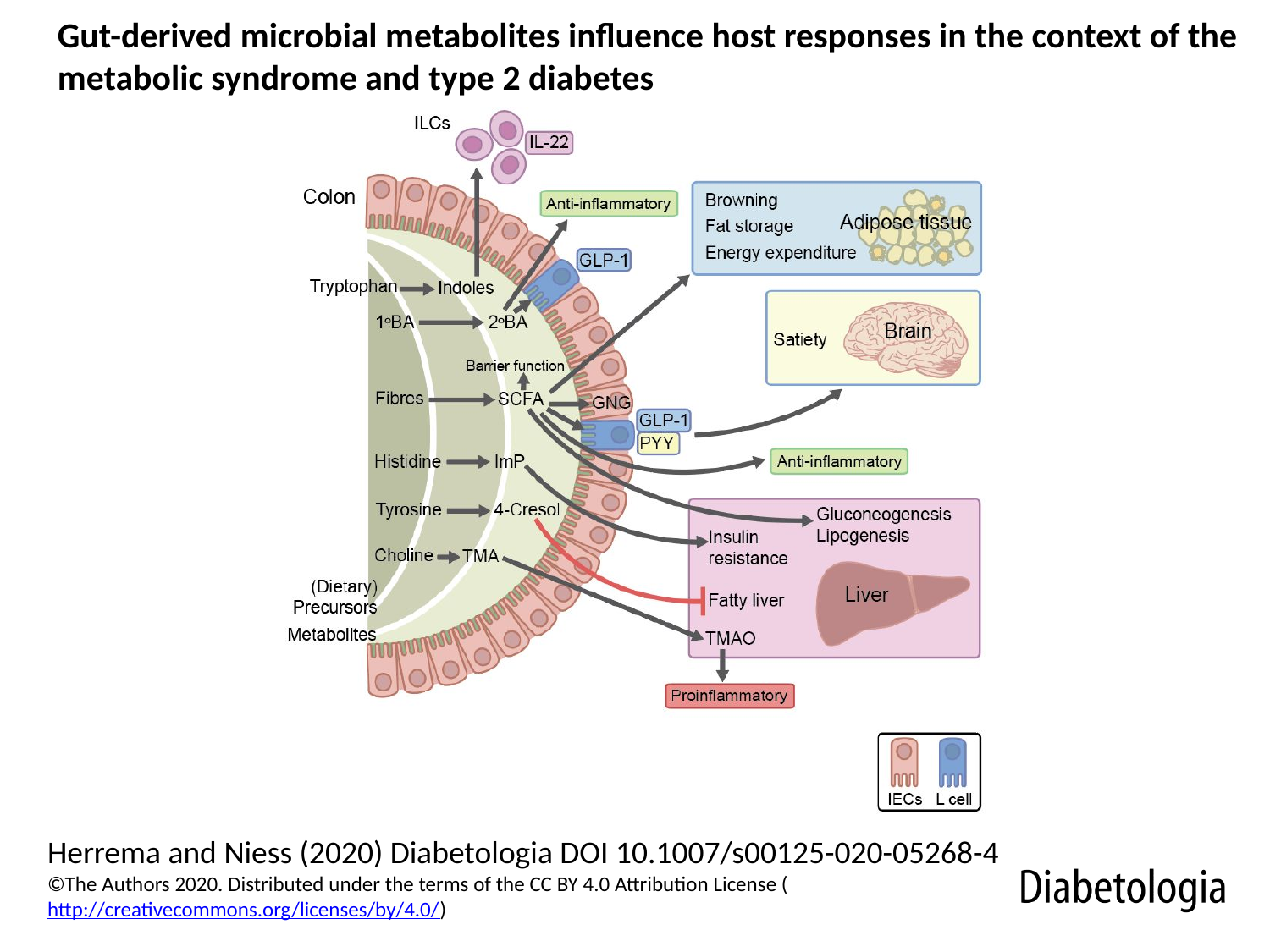

Gut-derived microbial metabolites influence host responses in the context of the metabolic syndrome and type 2 diabetes
Herrema and Niess (2020) Diabetologia DOI 10.1007/s00125-020-05268-4
©The Authors 2020. Distributed under the terms of the CC BY 4.0 Attribution License (http://creativecommons.org/licenses/by/4.0/)
